# Gene expression in mice with endothelium-specific telomerase knockout

**DOI:** 10.3389/fcell.2023.1295072

**Published:** 2023-12-07

**Authors:** Zhanguo Gao, Yongmei Yu, Yulin Dai, Zhongming Zhao, Kristin Eckel-Mahan, Mikhail G. Kolonin

**Affiliations:** ^1^ The Brown Foundation Institute of Molecular Medicine, University of Texas Health Science Center, Houston, TX, United States; ^2^ Center for Precision Health, Cancer Genomics Core, University of Texas Health Science Center, Houston, TX, United States

**Keywords:** endothelial, senescence, telomerase, TERT, knockout, mouse, gene expression

## 1 Introduction

Endothelial cells (EC), lining the vasculature, serve essential functions fundamental for normal physiology of every organ and the organism as a whole. EC are the first line of exposure to toxic xenobiotics as well as endogenously generated molecules causing cell damage and aging. Cell senescence, the irreversible arrest of cell proliferation caused by organelle damage, is an underpinning of aging responsible for tissue changes leading to age-related diseases (Fossel et al., 2022). Cell senescence is characterized by senescence-associated gene expression, and pro-inflammatory senescence-associated secretory phenotype (SASP), which negatively affects the function of non-senescent cells (Gorgoulis et al., 2019; Yousefzadeh et al., 2021; Fossel et al., 2022). The role of EC senescence in aging and disease has remained insufficiently understood. Even in mice it has not been established how EC senescence underlies the pathophysiology of aging by affecting other components of the vascular system, the perivascular/smooth muscle cells, as well as the parenchyma. In distinct organs, EC are exposed to different microenvironmental pressures and may have different rates of replication and self-renewal. In organs undergoing constant remodeling, such as adipose tissues (AT) and skeletal muscle, high proliferative pressure on EC is expected to result in telomere attrition occurring sooner than in other organs. However, reactive oxygen species and other metabolism byproducts may also expedite senescence of EC irrespective of their proliferation in other organs, such as the brain. The resulting dysfunction of EC leads to conduit vessel disease and obstruction to flow, as well as rarefaction of the microvasculature. This may set the stage for cardiovascular diseases and neurodegeneration (Xu et al., 2022). Understanding the changes taking place in EC undergoing senescence in the brain and other organs is essential for the development of new approaches to intervene in metabolic and degenerative diseases.

A key protein protecting cells from senescence is Telomerase, an enzyme coded for by the *TERT* gene. TERT is required for telomere maintenance, protection from genotoxic stress, and mitochondrial function (Sahin and Depinho, 2010). TERT is active in stem cells but is turned off in somatic cells, which permits telomere erosion and cell aging. Telomere-independent functions of TERT have also surfaced and remain incompletely understood (Stewart et al., 2002; Romaniuk et al., 2019). In addition to global effects on nuclear transcriptome and physiology (Park et al., 2009), recent reports have revealed an important function of TERT in the mitochondria (Ale-Agha et al., 2021; Ait-Aissa et al., 2022). Evidence is accumulating that re-activation of telomerase can have beneficial anti-aging effects (Nazari-Shafti and Cooke, 2015). In mice, TERT gene therapy delays aging and increases longevity (Bernardes de Jesus et al., 2012; Jaijyan et al., 2022). In a clinically relevant study, induction of TERT decreased DNA damage activation and inflammatory signaling in human colon organoids from patients with inflammatory bowel disease (Chakravarti et al., 2021). Moreover, TERT gene therapy enhances learning pathway networks in human neurons (Shim et al., 2021).

The lack of studies on organ specificity of EC senescence mechanisms and repercussions is in part due to laboratory mice being inappropriate as a model to study replicative senescence (Eckel-Mahan et al., 2020). Laboratory mice are not an ideal model to access consequences of replicative senescence. Humans are born with telomeres in a 10–15 kb range and *TERT* is inactivated in humans postnatally. In contrast, mice of the commonly used C57BL/6 background are born with telomeres of over 50 kb and continue to express *TERT* in somatic cells (Kipling and Cooke, 1990). Thus, laboratory mice are more resistant to replicative senescence and stem cell depletion. Indeed, disruption of telomere function in EC has been shown to induce premature senescence (Barinda et al., 2020; Bloom et al., 2023). As we have previously reported, knockout (KO) of TERT in perivascular cells of *Pdgfra+* or *Pdgfrb+* lineage accelerates the onset of cell senescence in adipose tissue (AT) of mice fed high-calorie diet (HCD), which predisposes them to type-2 diabetes (Gao et al., 2020). Here, we have generated mice with TERT gene knock-out (KO) specifically in EC. We performed genomic analysis of EC from AT and skeletal muscle by both total RNA and single cell (sc) RNA sequencing (RNAseq). We also challenged the mice with HCD to determine its effect on EC senescence and function. Preliminary analysis of data deposited online is presented. Our results suggest that TERT has genome-wide telomere-independent effects on cell transcriptome and physiology (Fossel et al., 2022).

## 2 Materials and methods

**FIGURE 1:**
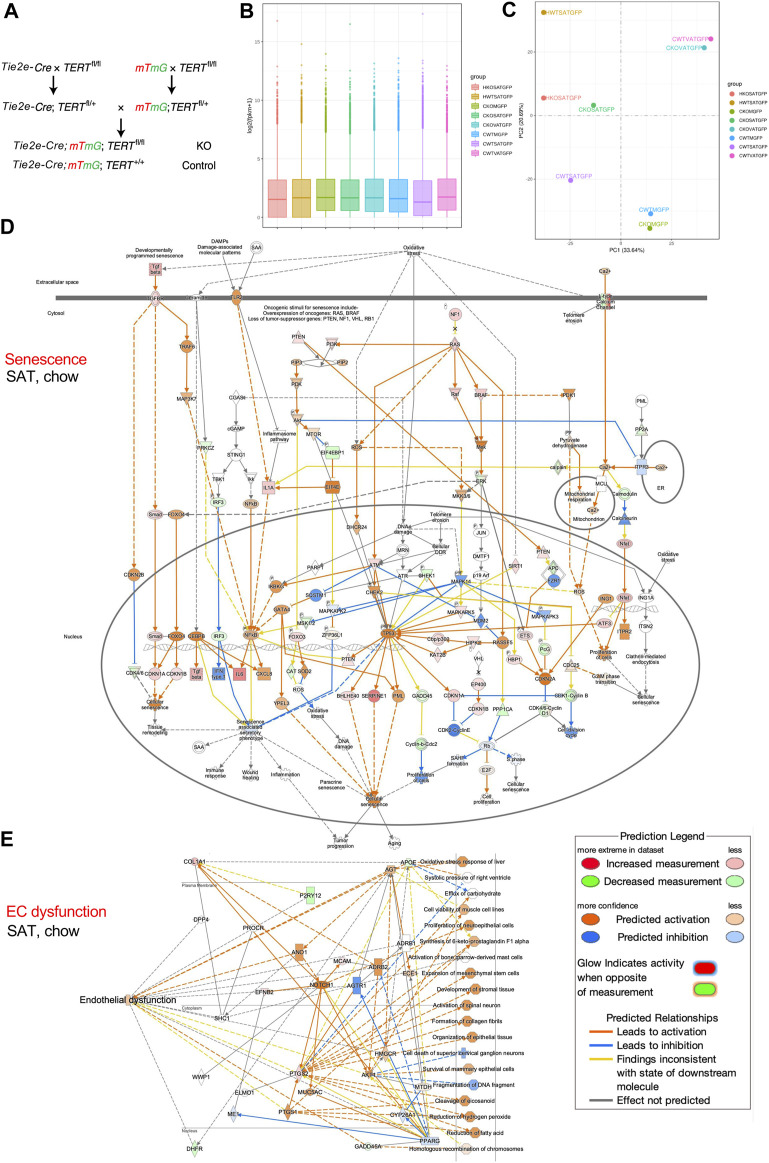
TERT knockout in mouse endothelial cells (EC). **(A)**, Breeding scheme to generate mice with mG+ and TERT-EC-KO (fl/fl) or WT (+/+) Tie2+ cells (EC) and other cells mT+. EC senescence and dysfunction caused by TERT loss was assessed in 8-month-old female mice fed chow (C) or HCD (H). Cells isolated from subcutaneous AT (SAT), intraperitoneal visceral AT (VAT), quadricep and gastrocnemius skeletal muscle (M) were subjected to FACS sorting to isolate mG+ cells for mRNA extraction. **(B)**, Gene expression distribution. X axis: mouse groups (also shown on the right). Parameters of box plots are indicated, including maximum, upper quartile, mid-value, lower quartile and minimum. **(C)**, Principal component analysis result (mouse groups shown on the right). **(D)**, IPA analysis focusing on senescence-related pathways identifies genes upregulated in mG+ cells from SAT of TERT-EC-KO mice fed chow compared to mG+ cells from SAT of WT mice fed chow. **(E)**, IPA analysis focusing on EC dysfunction-related pathways identifies genes upregulated in mG+ cells from SAT of TERT-EC-KO mice fed chow compared to mG+ cells from SAT of WT mice fed chow.

We crossed mice expressing Cre under the control of EC-specific Tie2e promoter (Kano et al., 2003) with TERT^fl/fl^ mice also carrying the mTmG reporter (Muzumdar et al., 2007) to generate mice with TERT gene knock-out (KO) specifically in EC (Figure 1A). Tie2e-cre; TERT^fl/fl^; mTmG (TERT-EC-KO) and control Tie2e-cre; TERT^+/+^; mTmG (WT) mice were fed HCD (D12451, 45 kcal% Fat, Research Diets) from 1 to 8 months of age. Cells isolated from subcutaneous AT (SAT), intraperitoneal visceral AT (VAT), as well as combined quadricep and gastrocnemius skeletal muscles (M), were subjected to FACS sorting to isolate mG+ (GFP+) cells (EC) for mRNA extraction by using protocols we described previously (Gao et al., 2018; Gao et al., 2021; Daquinag et al., 2022; Gao et al., 2022). Data quality control confirmed 2.3 × 10^7^ or more reads for all samples. At least 91.5% of reads for all samples were mapped to the mouse genome sequence database. To compare gene expression levels in the tissues, the distribution of gene expression levels and expected number of fragments per kilobase of transcript sequence per millions base pairs sequenced (FPKM) was assessed. Gene expression distribution was found comparable among all samples (Figure 1B). RNA-seq confirmed *TERT* gene expression loss in mG+ cells of TERT-EC-KO mice. In another experiment, we fed cohorts of TERT-EC-KO and control WT male mice with high-calorie diet from 2 to 7 months of age. Cells were then isolated from SAT and visceral VAT and subjected to single cell RNA sequencing (scRNA-seq) using methodology that we previously described (Gao et al., 2020). Single cell capture and library construction were performed with the Chromium Single Cell 3ʹ Reagent Kit v3.1. Barcoded single-cell gel beads were loaded onto Chromium Next GEM ChipG (PN-1000120). After running on 10X Chromium Single Cell Controller, gel beads-in-emulsion (GEMs) were generated. The barcoded and full-length cDNAs were produced after incubation of the GEMs and amplified via PCR. Library was qualified by Agilent Bioanalyzer 2,100 and quantified by real-time PCR on QuantStudio3. Sequencing was done with Illumina NextSeq 550 System using High Output Kit v2.5 (50,000 reads per cell). The Cell Ranger™ Single Cell Software Suite v.3.1.0 was used to perform bioinformatic analysis. The reads were aligned to the mouse transcriptome reference (mm10, Ensembl 93) with STAR (Dobin et al., 2013). Raw read count tables were analyzed using the Seurat (v3.1.1) pipeline (Butler et al., 2018) on R platform (3.5.2). FindVariableGenes was used to calculate the principal components. Cell clusters were identified using the Shared Nearest Neighbor (SNN) algorithm with a resolution parameter 0.8. UMAP clusters of cells were identified based on the first 10 principal components and feature plots were displayed with the log (raw read count +1) of gene/cell on UMAP.

## 3 Data analysis

Total RNA-seq was performed on mRNA from mG+ cells (EC) of TERT-EC-KO and control WT mice. Principal component analysis (PCA) was used to evaluate inter-sample differences. As expected, this revealed close similarity of gene expression in AT of WT and TERT-EC-KO mice and in skeletal muscle of WT and TERT-EC-KO mice (Figure 1C). HCD feeding resulted in a marked change of gene expression (Figure 1C). Notably, gene expression in SAT from TERT-EC-KO mice fed chow, compared to WT mice fed chow, was found to be more similar to that in SAT of mice fed HCD (Figure 1C).

We then performed Ingenuity Pathway Analysis (QIAGEN IPA) on FACS-sorted AT and muscle EC. Analysis of genes upregulated in mG+ cells of TERT-EC-KO mice demonstrated that a number of key genes implicated in cell senescence were induced in SAT (Figure 1D). This included *CDKN1A, CDKN1B, CDKN2A, CDKN2B, TP53*, as well as SASP genes *IL6* and *CXCL8*. Induction of pathways mediating senescence was also observed in VAT and skeletal muscle of TERT-EC-KO mice (data not shown). IPA analysis also revealed upregulation of genes specifically implicated in EC dysfunction, including *APOE, AGT, ANO1, MYC5AC,* and *NOTCH1* (Figure 1E). This was also apparent for VAT, and less so for skeletal muscle, of TERT-EC-KO mice (data not shown). IPA analysis of SAT from mice fed HCD revealed a markedly higher level of upregulation of senescence effectors, including *CHEK1, p19Arf*, and *CDKN2A* (Supplementary Figure S1A) in additional to upregulation of endothelial dysfunction pathways (Supplementary Figure S1B).

**FIGURE 2:**
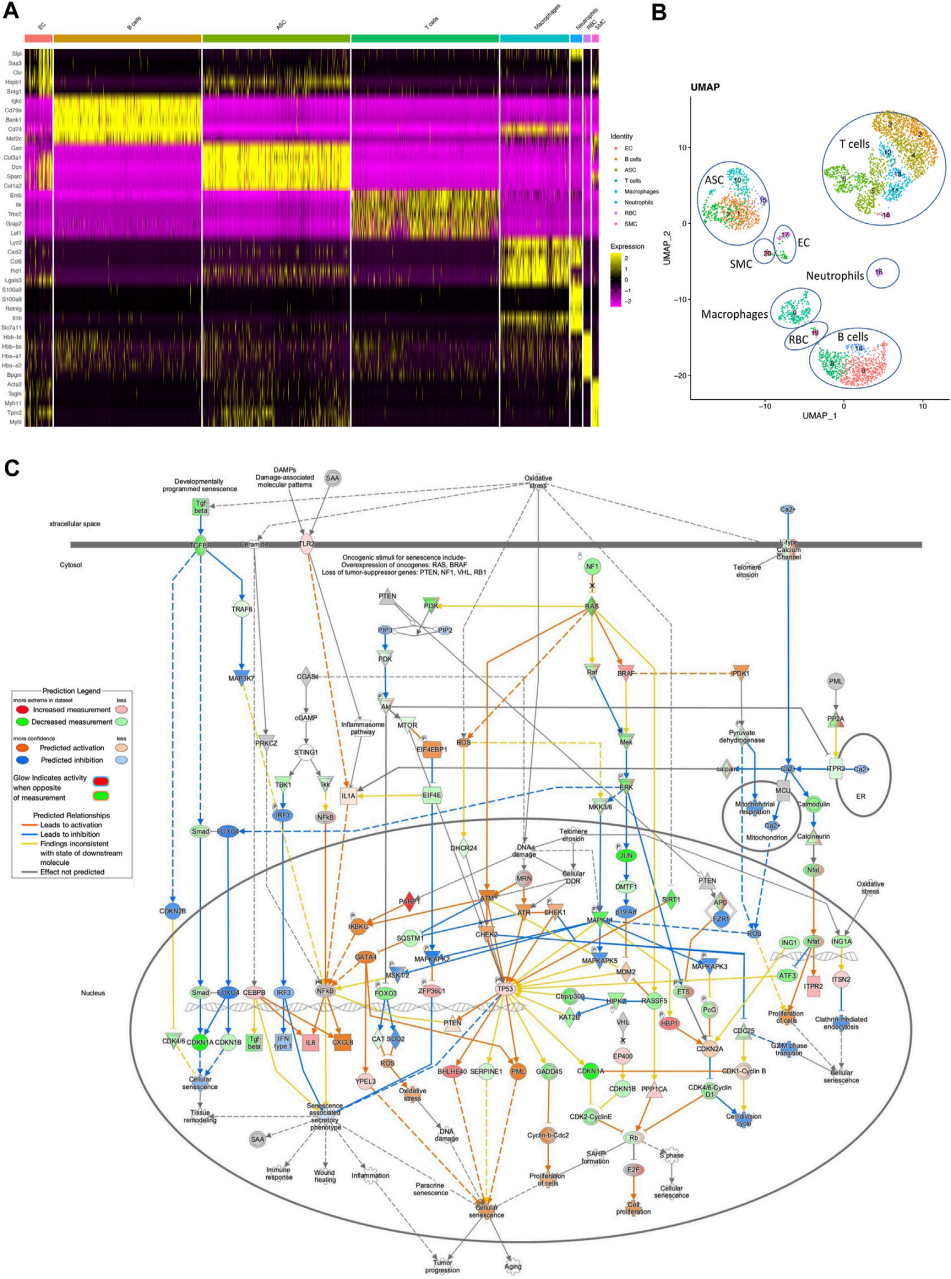
Single cell transcriptomics of adipose cells from EC Tert KO mice. TERT-EC-KO and WT 7-month-old male mice were fed HCD for 5 months prior to cells isolation from SAT and VAT and RNAseq. **(A)**, Integrated heatmap of combined RNAseq data with genes (left) identifying cell clusters designated on top. ACS, adipose stromal cells; RBC, red blood cells; SMC, smooth muscle cells. **(B)**, Regression UMAP clusters of combined KO and WT cells from SAT and VAT generated based on the first 10 principal components displayed with the log (raw read count +1) of gene/cell. **(C)**, Pathways upregulated in SAT EC of EC-TERT KO mice fed chow identified in scRNAseq data by IPA analysis focusing on senescence-related pathways.

Finally, we assessed scRNAseq data from AT of TERT-EC-KO and WT mice fed HCD. This analysis identified two sub-populations of EC, as well as smooth muscle cells (SMC), adipose stromal cells (ASC), as well as the distinct types of blood cells (Figures 2A, B). UMAP cluster analysis revealed that SAT of TERT-EC-KO mice had a markedly increased presence of certain T-cell subtypes, while the presence of one B-cell subtype was reduced (Supplementary Figure S2). IPA analysis of EC revealed higher expression of senescence-associated genes in EC of TERT-EC-KO AT (Figure 2C). These included *ATM, CHEK1, CHEK2, TP53, CDKN2A*, as well as SASP genes *IL6* and *CXCL8*.

The data, being consistent between total RNAseq and scRNAseq experiments, demonstrate that the TERT-EC-KO mice are an appropriate model of EC senescence. These mice can be used to further characterize consequences of endothelial senescence and develop models of aging/disease by subjecting these models to vascular injury insults.

## Data Availability

The datasets presented in this study can be found in online repositories. The names of the repository/repositories and accession number(s) can be found below: Total RNA-seq: GEO database via GSE239686 and single-cell RNA-seq: GSE239687.
