# Supplementary material for: Gene expression in mice with endothelium-specific telomerase knockout
Source: Front Cell Dev Biol. 2023 Dec 7;11:1295072. doi: 10.3389/fcell.2023.1295072 (PMC10755458; doi:10.3389/fcell.2023.1295072)
Supplement: Supplementary file 1 [file DataSheet1.PDF]

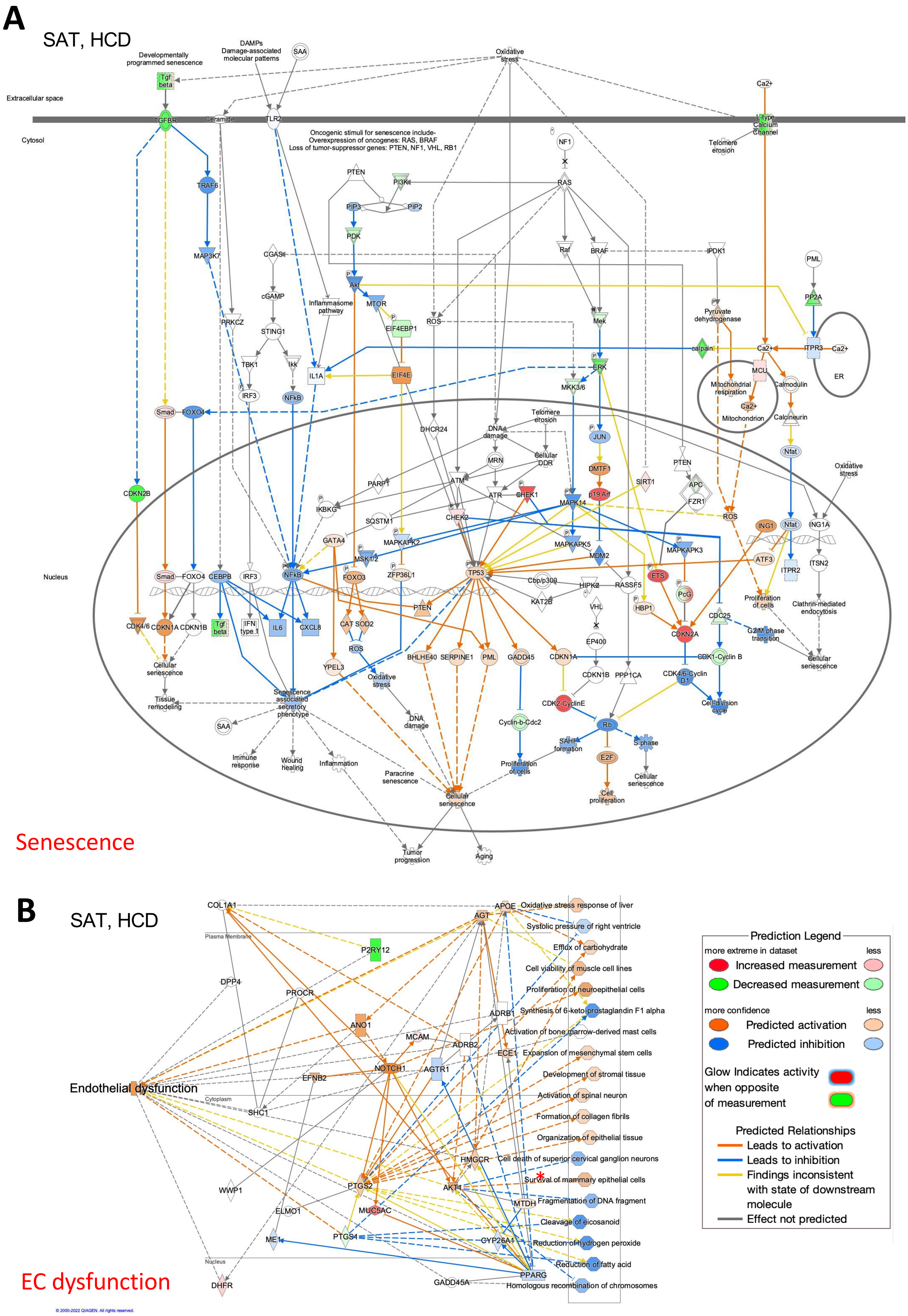

Supplementary Figure S1. TERT knockout in endothelial cells of mice fed high calorie diet. IPA analysis identifies genes upregulated in mG+ cells from SAT of EC-TERT-KO mice compared to mG+ cells from SAT of WT mice fed HCD. **A**, senescence-related pathways. **B**, EC dysfunction-related pathways.

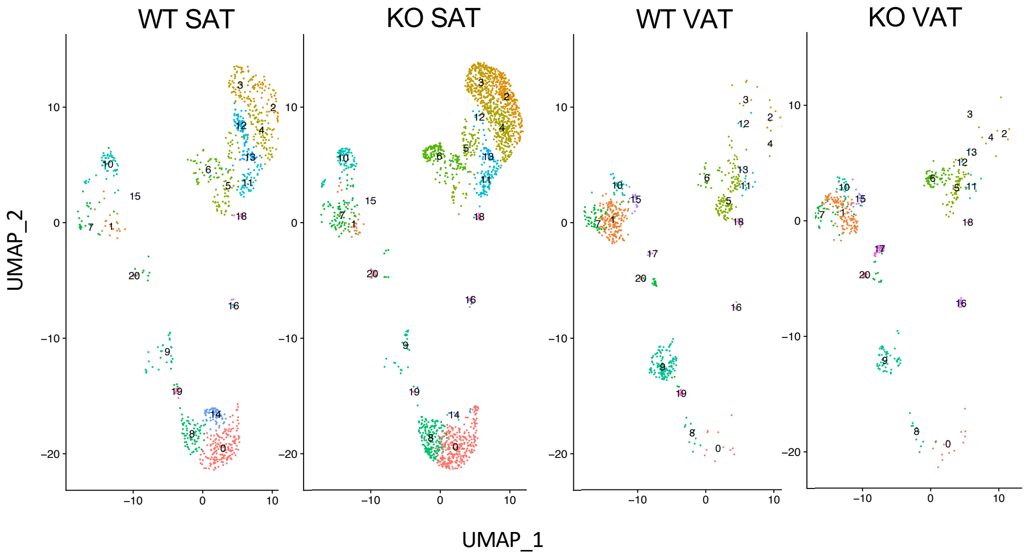

Supplementary Figure S2. Regression UMAP clusters of SAT and VAT cells from EC-TERT-KO and WT mice generated based on the first 10 principal components displayed with the log (raw read count +1) of gene / cell.
